# Fluconazole resistance among *Candida* species with special emphasis on *ERG11* gene mutations among *Candida tropicalis*

**DOI:** 10.3389/ffunb.2025.1695271

**Published:** 2026-01-12

**Authors:** Vidyavathi B. Chitharagi, Gowthami S., Mahadevaiah Neelambike Sumana, Morubagal Raghavendra Rao, Sowmya G. S., Yogeesh D. Maheshwarappa

**Affiliations:** Department of Microbiology, JSS Medical College and Hospital, JSS Academy of Higher Education and Research, Mysuru, India

**Keywords:** azole resistance, *Candida* isolates, *Candida tropicalis*, *ERG11* mutation, fluconazole resistance, polymerase chain reaction

## Abstract

**Introduction:**

Candidiasis, an opportunistic fungal infection, is increasingly caused by non-*albicans Candida* species that show reduced fluconazole susceptibility, mainly due to *ERG11* overexpression. This study aimed to identify *Candida* species, determine fluconazole resistance using VITEK 2 and disc diffusion methods, and detect *ERG11* gene mutations in *Candida tropicalis.*

**Methodology:**

A total of 410 clinical samples were included in this laboratory-based prospective study conducted at a tertiary care hospital in Mysuru. Fluconazole-resistant *Candida* species were identified using the Vitek-2 system and disc diffusion methods. The *ERG11* gene of fluconazole-resistant strains of *Candida tropicalis* was amplified by polymerase chain reaction (PCR) and subjected to high-resolution melt (HRM) analysis to detect A395T and C461T mutations.

**Results:**

A total of 410 *Candida* species were isolated from 410 clinical isolates during the study period, with 61% (250/410) from males and 39% (160/410) from females. Among the 410 isolates tested by Vitek-2, 29 (7.07%) were resistant to fluconazole, with the majority being *C. tropicalis* (51.7%). Of the 15 C*. tropicalis* isolates tested, A395T and C461T mutations in the *ERG11* gene were detected in 6 isolates. These isolates showed high minimum inhibitory concentrations (MICs) to azoles. Discrepancies between Vitek-2 and PCR findings likely reflect the multifactorial nature of fluconazole resistance and the presence of resistance mechanisms beyond the targeted *ERG11* mutations.

**Conclusion:**

The study concludes that antifungal susceptibility testing (AST) using Vitek-2 is a preferred method in the laboratory for identifying *Candida* species and performing susceptibility testing due to its ease of use and cost-effectiveness. Disc diffusion can be utilized in resource-limited settings to guide treatment, while PCR and newer molecular methods offer valuable opportunities for researching different mechanisms and mutations responsible for fluconazole resistance, a widely used antifungal for treatment and prevention.

## Introduction

Candidiasis is the most common fungal disease in humans, affecting the mucosa, skin, nails, and internal organs. It is caused by the genus *Candida*. It has a wide range of clinical spectra, including both deep and superficial infections. It is mainly found as an opportunistic infection in immunocompromised individuals and rarely as the primary disease. The genus *Candida* comprises 163 species, but a few of these species cause human infections, including *C. albicans*, *C. tropicalis*, *C. krusei*, *C. glabrata*, *C. auris*, *C. haemulonii*, *C. parapsilosis*, *C. lusitaniae*, *C. kefyr*, *C. rugosa*, *C. dubliniensis*, and *C. viswanathii*. Among all these species, *C. albicans* is found to be the most potent pathogenic organism responsible for about 40% of infections (Ying et al., 2013; Ying et al., 2015).

The *Candida* species are found as commensals of the gastrointestinal tract and the female genital tract, particularly in higher concentrations in the vagina during pregnancy. Several predisposing factors contribute to the development of both superficial and deep-seated candidiasis. These factors act either by altering the balance of the body’s normal flora or by lowering the host’s resistance (Nnadi et al., 2012; Sardari et al., 2019). Fluconazole, a widely used azole derivative, offers proper water solubility, extensive bioavailability, and long half-life for once-daily dosing. However, non-albicans species like *C. tropicalis, C. parapsilosis* and other species exhibit resistance to fluconazole and *C. glabrata* shows increased resistance to fluconazole, while *C. krusei* displays intrinsic resistance to fluconazole (Bart-Delabesse et al., 1993; Fadda et al., 2010; Wang et al., 2019).

Fluconazole acts by inhibiting lanosterol 14α-demethylase, an enzyme encoded by the *ERG11* gene that plays a crucial role in ergosterol biosynthesis, essential for fungal cell membrane integrity. Resistance to fluconazole may occur through various mechanisms, including mutations in the *ERG11* gene leading to amino acid substitutions that reduce fluconazole binding affinity, and overexpression of the *ERG11* gene resulting in increased production of the enzyme. Other resistance mechanisms include efflux pump overactivity. These molecular changes enable continuous synthesis of ergosterol, maintaining cell wall integrity and contributing to fluconazole resistance in *Candida* species. Continuous usage of fluconazole as prevention has been implicated as a cause of resistance (Bart-Delabesse et al., 1993; Sem et al., 2024).

Additionally, fluconazole-resistant *Candida* species exhibit variable susceptibility due to mutations in the *ERG11* gene, which may contribute to pan-azole resistance. Hence, this study aimed to identify *Candida* species, determine fluconazole resistance using VITEK 2 and disc diffusion methods, and detect *ERG11* gene mutations in *Candida tropicalis* by polymerase chain reaction (PCR).

## Methodology

### Sample collection/study design

The laboratory-based prospective experimental study was conducted at a Tertiary care Hospital in Mysuru, Karnataka, over one year. The samples were collected using a convenient sampling method. The study included 410 clinical samples (urine, pus, endotracheal aspirates, ear swabs, and sputum), yielding the growth of *Candida* species, while samples yielding the growth of yeasts other than *Candida* were excluded. The study was approved by the Institutional Ethics Committee of JSS Medical College and Hospital, Mysuru, South India, and verbal informed consent was obtained from patients or patient attendees before sample collection. All data were anonymized before analysis.

### Isolation of *Candida* species

The collected clinical samples were subjected to microscopic examination (Gram’s staining and KOH mount) and cultured on Sabouraud’s dextrose agar (SDA) (HiMedia Laboratories, Pvt. Ltd., Mumbai, India) and Potato dextrose agar (PDA) (HiMedia Laboratories Pvt. Ltd., Mumbai, India), incubated at appropriate conditions (37°C for 24 hours, aerobically). The suspected colonies were identified using both conventional (Gram’s staining) and an automated identification system (Vitek-2 compact system by bioMérieux, France) (Sandven, 1999; Vandeputte et al., 2005).

### Speciation of *Candida* isolates

The germ tube test, sugar assimilation test and cornmeal agar were used to differentiate between *C. albicans* and non-albicans species.

### Antimicrobial susceptibility testing

The antimicrobial susceptibility test (AST) was also performed using an automated identification system (Vitek-2 compact system by bioMérieux, France) with the AST-YS08 panel of drugs. The AST results were also tested simultaneously by the Kirby-Bauer disk diffusion method. And the results were compared. All tests were performed, and the results were interpreted in accordance with the latest Clinical and Laboratory Standards Institute (CLSI-M44A) guidelines (Vandeputte et al., 2005; Cilo and Ener, 2021).

For automated testing, the Vitek-2 Compact System was used with the AST-YS08 yeast susceptibility cards containing predefined concentrations of antifungal agents, including fluconazole, voriconazole, amphotericin B, caspofungin and flucytosine. Fresh yeast suspensions were prepared in sterile saline and adjusted to a McFarland standard of 1 to 1.5, as recommended by the manufacturer. The inoculated cards were loaded into the Vitek-2 compact system, and results were automatically interpreted by the system software according to Clinical and Laboratory Standards Institute (CLSI M44-A) guidelines.

The disc diffusion method was carried out on cation-adjusted Mueller-Hinton agar, as per CLSI recommendations. A standardized yeast suspension (0.5 McFarland) was inoculated (lawn culture) onto the agar surface, and fluconazole (25 µg) discs (HiMedia Laboratories Pvt. Ltd., Mumbai, India) were placed aseptically. Plates were incubated at 35°C for 24 hours, and zone diameters were measured in millimeters and interpreted as susceptible or resistant according to CLSI M44-A interpretive criteria (Jeddy et al., 2011).

To assess the accuracy of the test results, internal quality control examinations for Disc diffusion were performed on culture media and antifungal discs (HiMedia Laboratories Pvt. Ltd.) using *Candida albicans* ATCC (American Type Culture Collection) 90028. The Quality control examination for pathogen identification and antimicrobial susceptibility testing by the Vitek-2 compact system was also routinely conducted following standard operating protocols guided by CLSI, using *Candida albicans* ATCC (American Type Culture Collection) 90028.

### Fluconazole resistance gene detection by polymerase chain reaction

#### Nucleic acid extraction

The nucleic acid (DNA) was extracted from 24-hour-old fresh *Candida* cultures (grown on an SDA medium) using the Qiagen DNA Mini Kit (Qiagen, Hilden, Germany) following the manufacturer’s protocol. The extracted DNA was subjected to Real-time polymerase chain reaction (RT-PCR) for the detection of the fluconazole-resistant ERG11 gene and subsequently analyzed using high-resolution melt (HRM) analysis to identify A395T and C461T mutations in the ERG11 gene (Tan et al., 2015; Paul et al., 2021).

#### Real-time polymerase chain reaction for *ERG11* gene detection

Real-time PCR (RT-PCR) and High-Resolution Melt (HRM) analysis were performed for ERG11 gene detection and mutation identification. The primers used in this study were adopted from Paul et al. (2021), who originally designed and validated these primers for the rapid detection of ERG11 polymorphisms in *Candida tropicalis* (Paul et al., 2021). The same primer sequences were employed in the present study for HRM analysis targeting the A395T and C461T mutations. The ERG11 gene sequence of *C. tropicalis* (accession number MYA3404) was used as the reference sequence. The specificity of the primers was further confirmed through in silico BLAST analysis against fungal, bacterial, and human genome databases to ensure minimal non-specific amplification. Primer sequences and expected amplicon sizes are provided in **Table 1**.

**Table 1:** Primer’s sequence.

Analysis	Mutation	Primer	Sequence	Product
HRM	A395T &C461T	Forward	5^1^-ACTCCTGTTTTAAAGGTGT-3^1^	131
		Reverse	3^1-^ACTTCTTCTCTGATCTTGGAACA-5^1^	

HRM-High-Resolution Melting analysis. Mutations A395T and C461T occur within the amplified ERG11 gene region but are not located within the primer sequences; therefore, no residues in the primer sequences differ from the wild-type, and none are bolded.

PCR reactions were performed in a total volume of 25 µl containing 12.5 µl 2× QIAGEN Type-it HRM PCR master mix, 0.5 µM each of forward and reverse primers (prepared from 10 mM primer stocks), variable concentrations of dNTPs as specified by the manufacturer, 1× PCR buffer, and 50 ng of template DNA. Details of all reagent concentrations, including dNTPs, MgCl2, and polymerase units, are specified according to the QIAGEN Type-it HRM PCR Kit protocol to enable assay reproducibility. PCR performed using QIAGEN Rotor-Gene Q instrument.

The PCR cycling conditions consisted of an initial denaturation at 95°C for 5 minutes, followed by 40 cycles of denaturation at 95°C for 10 seconds, annealing at optimized temperatures for 30 seconds, and extension at 72°C for 30 seconds, followed by a final extension at 72°C for 5 minutes. HRM analysis was performed immediately after PCR to identify single-nucleotide polymorphisms. Data were analyzed using real-time normalized melt curves. The primers used for amplifying the *ERG11* gene were adopted from Paul et al. (2021), who previously designed and validated these primers for rapid detection of the A395T and C461T mutations in *Candida tropicalis* (Paul et al., 2021). Although direct sequencing of PCR products to confirm the mutations was not performed in this study, the reliability of the assay was supported by the use of these validated primers and by in silico analysis to minimize non-specific amplification from bacterial and human DNA. Additionally, PCR assay conditions were optimized for specificity and reproducibility using positive and negative control strains previously characterized by phenotypic fluconazole susceptibility. These measures provide reasonable confidence in the assay’s ability to detect the targeted *ERG11* mutations.

#### Statistical analysis

The data obtained in this study were expressed as numbers and percentages. Comparative analysis of fluconazole resistance in *Candida tropicalis* isolates, as determined by the Vitek-2 system, disc diffusion method, and PCR assay, was performed using SPSS (Statistical Package for Social Sciences) statistical software 20. A p-value of less than 0.05 was considered statistically significant, indicating meaningful differences among the methods in detecting resistance.

## Results

**Figure 1:**
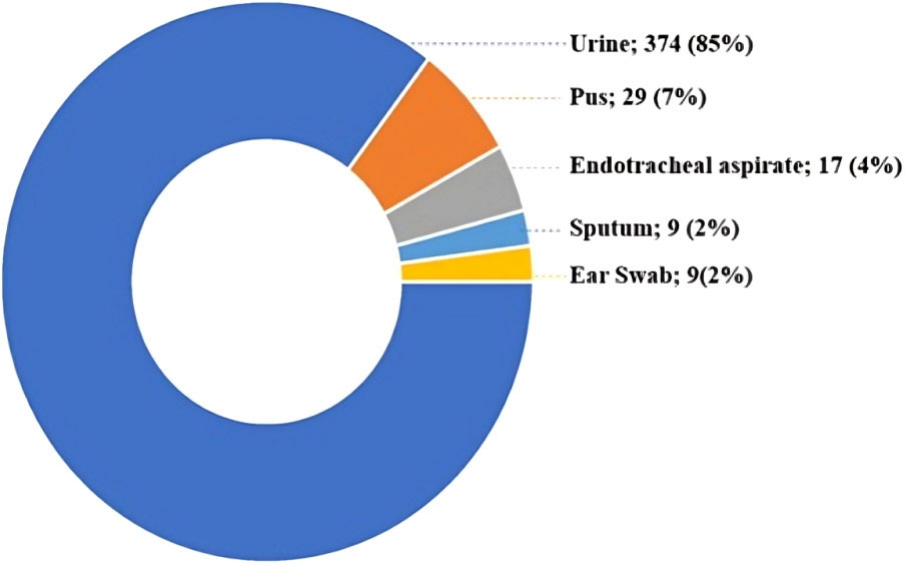
Distribution of clinical specimens yielding *Candida* isolates.

During the study period, 410 *Candida* species were isolated and identified using an automated identification system (Vitek-2 compact system by bioMérieux, France). The maximum number of *Candida* isolates was received from males (N = 250, P = 61%), followed by females (N = 160, P = 39%). Among the total *Candida* isolates, the majority were isolated from urine samples, followed by pus, endotracheal aspirates, and the least isolates were from ear swabs and sputum, as shown in **Figure 1**.

**Figure 2:**
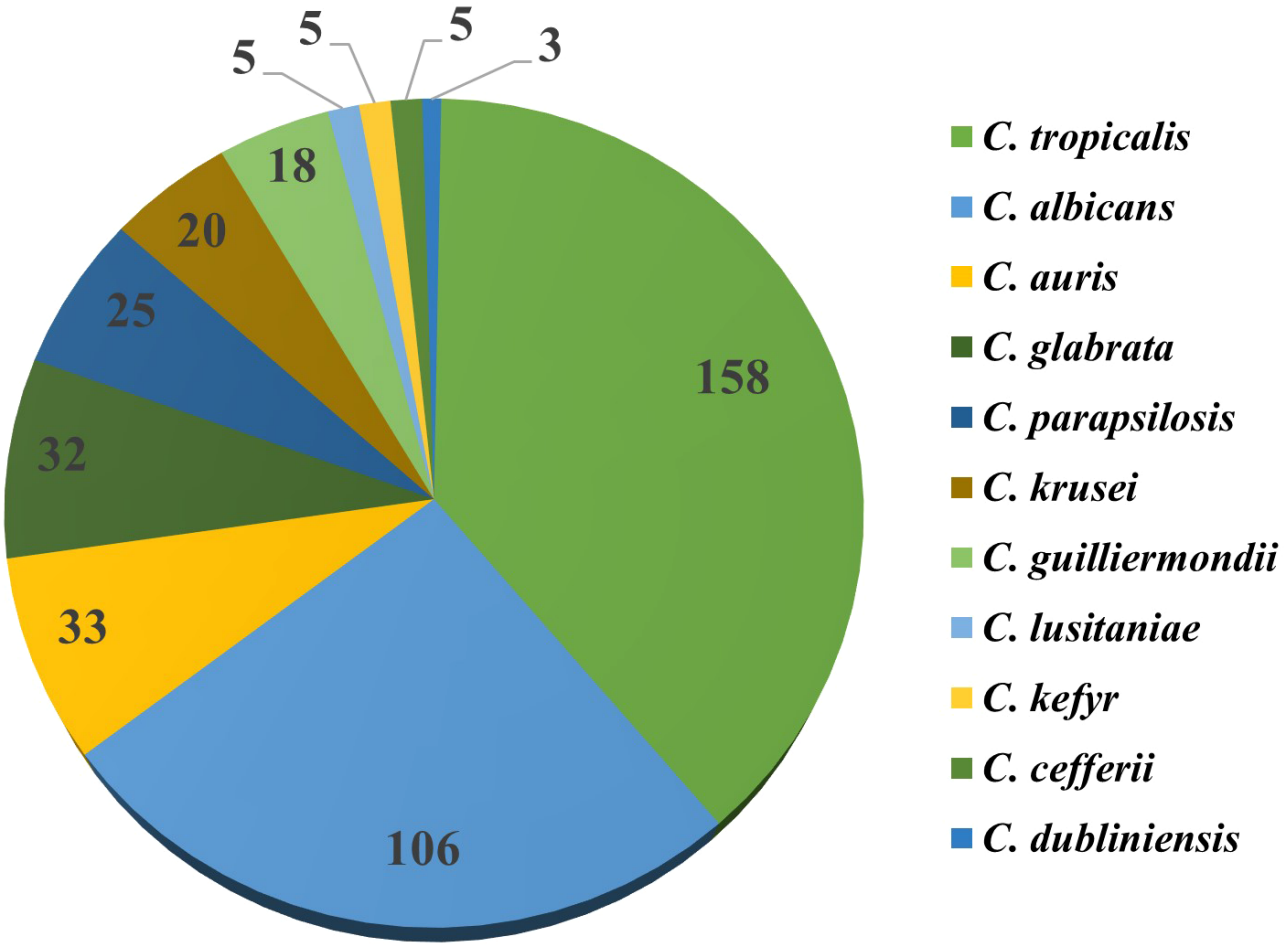
Species wise distribution of *Candida* isolates identified using the Vitek-2 compact system.

Among the 410 *Candida* isolates identified in this study, *Candida tropicalis* was the predominant species (158 isolates, 39%), followed by *C. albicans* (106 isolates, 26%). Other species identified include *C. auris* (33 isolates;8%), *C. glabrata* (32 isolates; 8%), and *C. parapsilosis* (25 isolates;6%). Less commonly isolated species included *C. krusei*, *C. guilliermondii*, *C. lusitaniae*, *C. kefyr*, *C. cefferii*, and *C. dubliniensis* (each ≤5%). The detailed species distribution is presented in **Figure 2**.

### Antimicrobial susceptibility pattern of *Candida* isolates by Vitek-2 compact system

According to the AST results from the Vitek-2 system, out of the 410 *Candida* isolates tested, 49 showed resistance to different antifungal agents. Among these, 29 (Excluding *C, auris*, which is intrinsically resistant to fluconazole) were resistant to fluconazole. Specifically, *C. tropicalis* accounted for 15 (51%), *C. albicans* for 3 (10%), *C. parapsilosis* for 3 (10%), *C. dubliniensis* for 3 (10%), *C. guilliermondii* for 3 (10%), *C. cefferii* for 1 (3%), and *C. lusitaniae* for 1 (3%) of the resistant isolates.

### Comparative analysis of fluconazole susceptibility between the Vitek-2 system and the disc diffusion method

**Figure 3:**
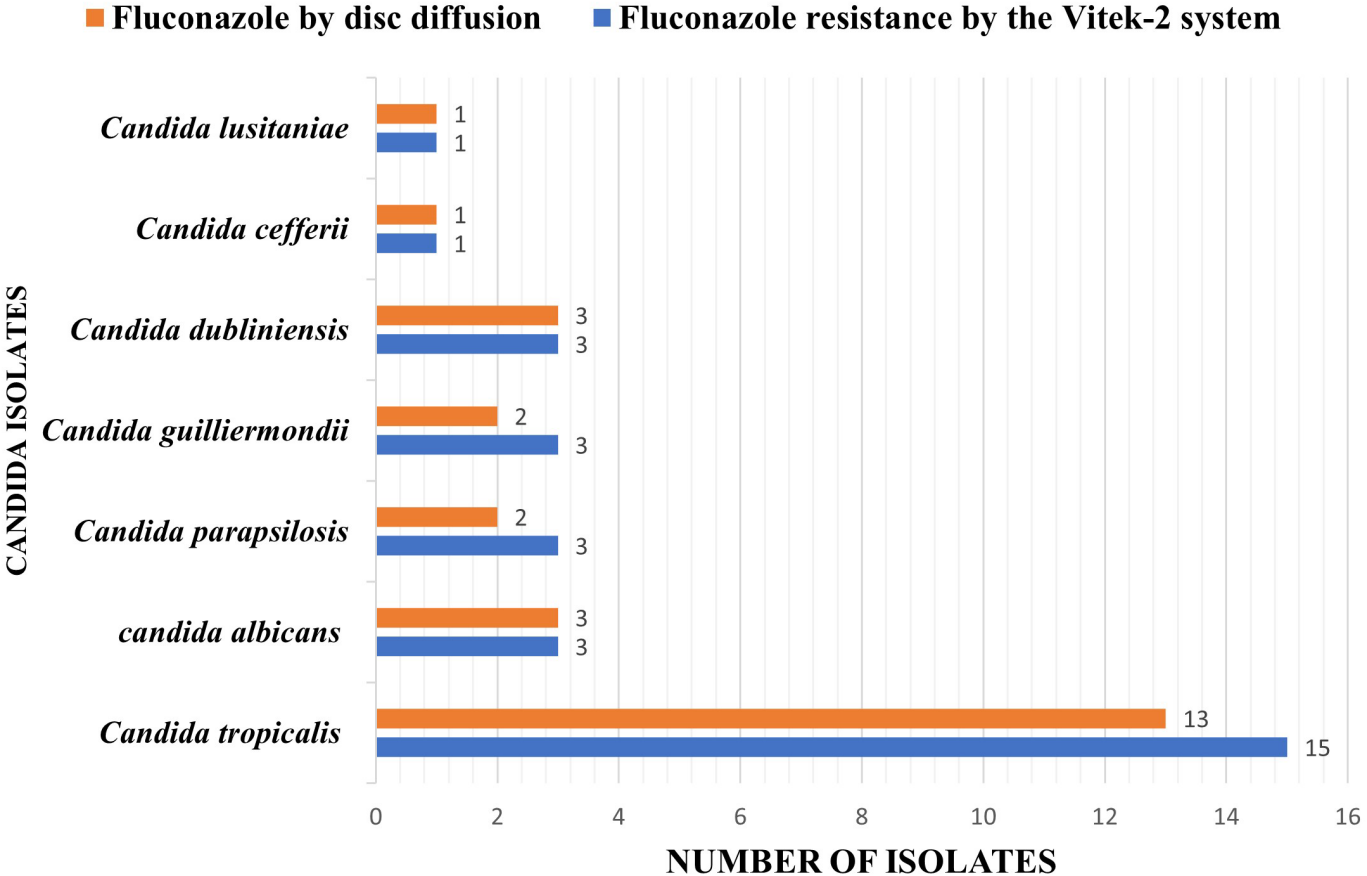
Comparative analysis of fluconazole susceptibility between the Vitek-2 system and the disc diffusion method.

A total of 29 fluconazole-resistant *Candida* isolates, as determined by the Vitek-2 system, were subjected to a disc diffusion test to determine their susceptibility to fluconazole. Among these isolates, 25 were found to be resistant to fluconazole by disc diffusion. Specifically, *C. tropicalis* accounted for 13 (87%), *C. albicans* for 3 (100%), *C. parapsilosis* for 2 (67%), *C. dubliniensis* for 3 (100%), *C. guilliermondii* for 2 (67%), *C. cefferii* for 1 (100%), and *C. lusitaniae* for 1 (100%) of the resistant isolates, as depicted in **Table 2**, **Figure 3**.

**Table 2:** Concordance between the Vitek-2 system and the disc diffusion method.

Sl, no	Candida isolates	Fluconazole resistance by the Vitek-2 system	Fluconazole by disc diffusion	Concordance between Vitek-2 and the disc diffusion method
1	*Candida tropicalis*	15	13	87%
2	*candida albicans*	3	3	100%
3	*Candida parapsilosis*	3	2	67%
4	*Candida guilliermondii*	3	2	67%
5	*Candida dubliniensis*	3	3	100%
6	*Candida cefferii*	1	1	100%
7	*Candida lusitaniae*	1	1	100%
Total		29	25	

Among the 410 *Candida* isolates tested for fluconazole susceptibility, 29 (7.1%) were resistant as determined by the Vitek-2 system. The disc diffusion method identified 25 resistant isolates, showing an overall concordance of 86% with the Vitek-2 results. The species-wise agreement ranged from 67% to 100%, with lower concordance observed for *C. parapsilosis* and *C. guilliermondii*. The overall sensitivity, specificity, positive predictive value, and negative predictive value of the disc diffusion method were 86.2%, 100%, 100%, and 99%, respectively. Cohen’s kappa value of 0.92 indicated almost perfect agreement between the two methods, and McNemar’s test (χ² = 2.25, p = 0.13) showed no statistically significant difference, as provided in **Table 3**, confirming the reliability of the disc diffusion method in detecting fluconazole resistance among *Candida* isolates.

**Table 3:** Statistical comparison of fluconazole susceptibility results obtained by Vitek-2 and disc diffusion methods.

Parameter	Formula/Statistical test	Result	Interpretation
Sensitivity	TP/(TP + FN)	86.2%	Disc diffusion correctly identified 86% of fluconazole-resistant isolates compared with Vitek-2
Specificity	TN/(TN + FP)	100%	Excellent ability to rule out false resistance
Positive Predictive Value (PPV)	TP/(TP + FP)	100%	Vitek-2 confirmed all resistant results by disc diffusion
Negative Predictive Value (NPV)	TN/(TN + FN)	99%	Nearly all susceptible results were true negatives
Overall Agreement	(TP + TN)/Total	99%	Excellent concordance between the two methods
Cohen’s Kappa (κ)	–	0.92	Almost perfect agreement between Vitek-2 and disc diffusion
McNemar’s Test	χ² =2.25, p=0.13	–	No statistically significant difference between the two methods
Species-wise Concordance	–	67–100%	Lower concordance observed for *C. parapsilosis* and *C. guilliermondii*

True Positive (TP): Isolates resistant by both Vitek-2 and disc diffusion methods (25), False Negative (FN): Isolates resistant by Vitek-2 but susceptible by disc diffusion (4), False Positive (FP): Isolates susceptible by Vitek-2 but resistant by disc diffusion (0), True Negative (TN): Isolates susceptible by both methods (381). Vitek-2 results were considered the reference standard. Total *Candida* isolates tested = 410 (29 resistant and 381 susceptible).

### Molecular detection of *ERG11* gene mutations in fluconazole-resistant *Candida tropicalis*

**Figure 4:**
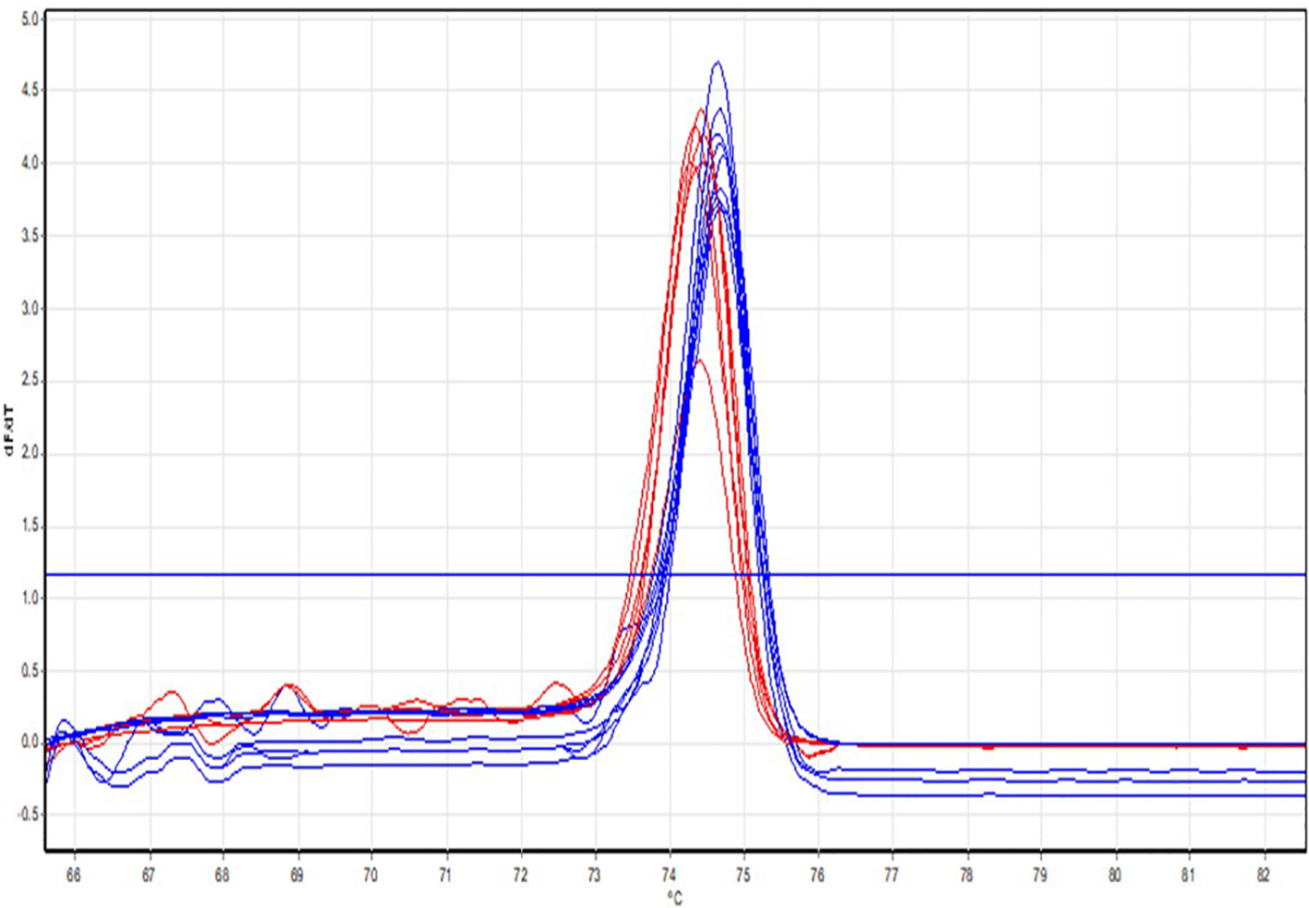
HRM normalisation curves. Normalised melt curves show two distinct melting profiles generated from the ERG11 amplicons. Isolates represented by the blue curves demonstrate a higher melting temperature (Tm), consistent with the wild-type ERG11 sequence. In contrast, isolates represented by the red curves exhibit a left-shifted melt transition with a lower Tm, indicating sequence variation associated with ERG11 point mutations. The curve separation reflects differences in amplicon stability caused by nucleotide substitutions; mutant isolates (red) denature earlier than wild-type isolates (blue).

**Figure 5:**
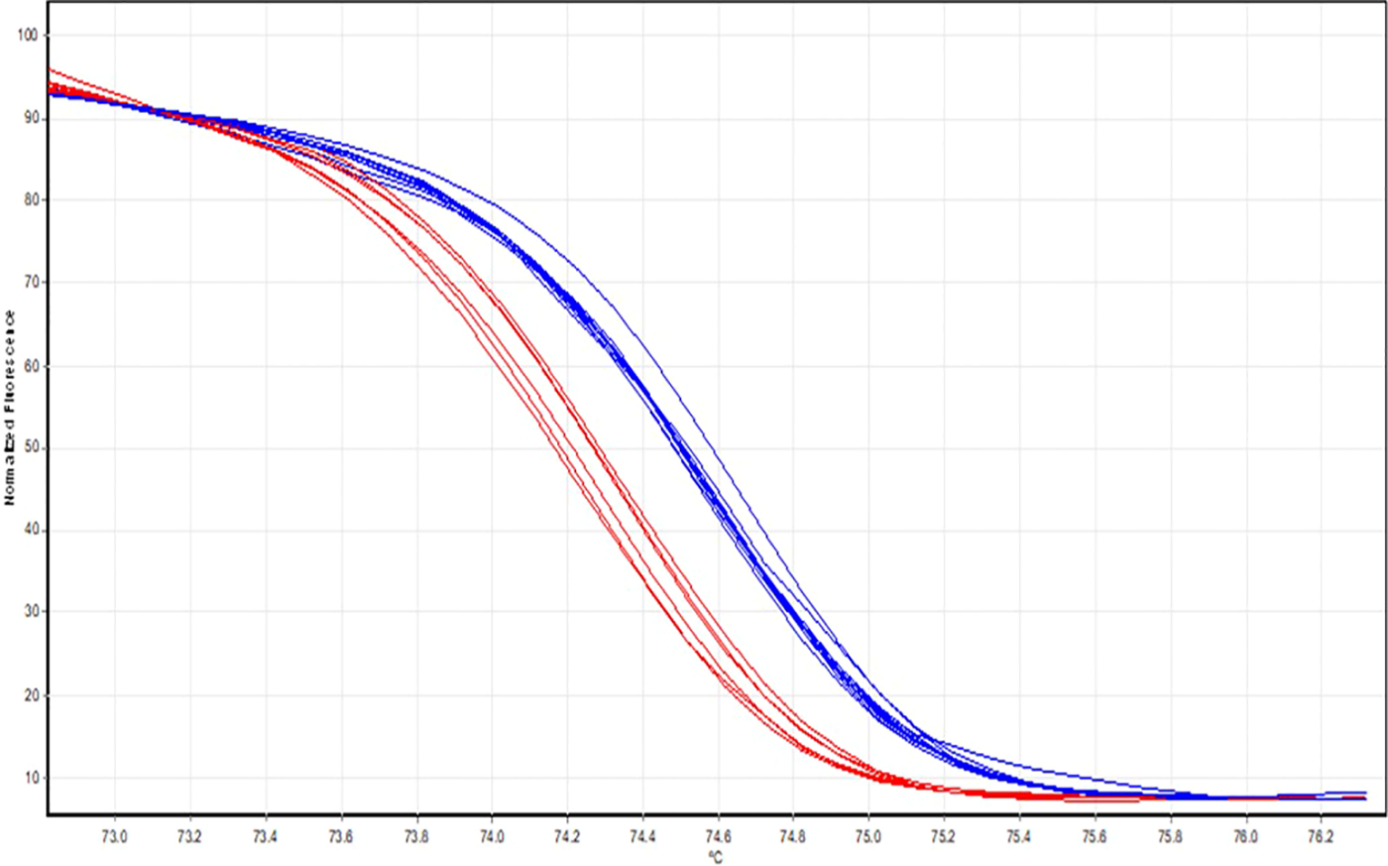
HRM differential plots. Differential melting plots (dF/dT) display distinct peak positions that separate ERG11 mutant and wild-type isolates. The blue cluster corresponds to wild-type isolates, which show a higher-temperature melting peak. The red cluster shows a lower-temperature peak, indicating the presence of ERG11 mutations (A395T and/or C461T). The shift in peak position and the formation of separate clusters directly indicate sequence alterations within the ERG11 gene. Mutation-positive isolates consistently show lower and left-shifted peaks.

The 15 *Candida tropicalis* isolates identified as fluconazole-resistant by the Vitek-2 system were further subjected to High-Resolution Melting (HRM) analysis for the detection of *ERG11* gene mutations. HRM analysis revealed the presence of *ERG11* mutations (A395T and C461T) in 6 isolates, as shown in **Figure 4**. Notably, both A395T and C461T mutations co-occurred in all six isolates. The HRM normalization and differential plots are depicted in **Figures 4**, **5**, respectively.

As presented in **Table 4**, among the 15 C*. tropicalis* isolates identified as fluconazole-resistant by the Vitek-2 system, 13 (87%) were confirmed resistant by the disc diffusion method, and 6 (23%) showed *ERG11* gene mutations (A395T and C461T) by PCR. This difference may be attributable to the presence of alternative resistance mechanisms that were not assessed in this study. Fluconazole resistance in *Candida* species can involve multiple molecular pathways beyond *ERG11* gene mutations, including overexpression of efflux pumps (e.g., *CDR1*, *MDR1*), alterations in ergosterol biosynthesis genes other than *ERG11*, and biofilm formation, all of which can contribute to azole resistance phenotypes.

**Table 4:** Comparative analysis of *C. tropicalis* fluconazole resistance results of Vitek-2 system, disc diffusion and PCR.

Method	Fluconazole-resistant isolates	Percentage	Sensitivity (%)	Specificity (%)	PPV (%)	NPV (%)	P value
Vitek-2 system	15	100	Reference	Reference	Reference	Reference	–
Disc diffusion test	13	87	86.7	100	100	88.9	0.001*
PCR (*ERG11* mutation)	6	23	40.0	100	100	55.6	0.001*

*represent significant association.

## Discussion

Candidiasis is an opportunistic infection that causes mucosal, respiratory, and urinary tract infections, and it is mostly caused in immunosuppressed/immunocompromised patients as a secondary infection. Candidal infections are treated with azoles. There is an increased resistance to azoles in the *Candida* species, to understand the mechanism of resistance and rapid development, which is reliable resistance detection is crucial. Various factors contribute to the development of azole resistance in *Candida* species; the ERG11 mutation in the coding sequence is directly related to the resistance against different azole drugs in the clinical setting.

Most commonly, *Candida* species are predominantly isolated from females, followed by males (Kim et al., 2016; Shaik et al., 2016; Sailaja and Prasad, 2019). However, in our study, most *Candida* species were isolated from males followed by females, with a male-to-female ratio of 1.5:1. A similar pattern was observed in a study conducted by Lata R Patel et al. in North India, which reported a male preponderance with an overall male-to-female ratio of 2:1 and another study by Saimandir et al. also found a higher incidence of *Candida* infections in males, reporting a male-to-female ratio of 1.7:1 (Patel et al., 2012; Marak and Dhanashree, 2018). The male predominance could be influenced by specific demographic and clinical factors such as diabetes, urinary catheters, and other underlying health conditions that predispose them to *Candida* infections. Additionally, the study includes fewer vaginal specimens; this could also be a reason for male predominance in *Candida* isolation.

In this study, the majority of the *Candida* species (N = 374, P = 85%) were isolated from urine samples. This finding aligns with studies conducted by Munmun B. Marak et al. in South India, which isolated 39 (43.33%) *Candida* isolates from urine samples, and Umamaheshwari S et al., who isolated 438 (58.32%) *Candida* species from urine samples. This similarity in findings underscores the prevalence of Candiduria (Chongtham et al., 2022; S and Sumana, 2023). In contrast to our findings, Urvashi Chongtham et al. in North India isolated *Candida* species predominantly from sputum samples, accounting for 43 (43%) isolates, and Vignesh Kanna B et al. in South India isolated *Candida* species predominantly from vaginal swabs. These variations may be attributed to regional differences in patient populations and healthcare practices, which influence the types of specimens collected. the preference for urine samples in our setting over vaginal swabs might be influenced by the higher rate of *Candida* detection in urine (Vignesh Kanna et al., 2017; Seyoum et al., 2020).

In this study site, *C. tropicalis* was predominantly isolated, with 158 isolates (39%), followed by *C. albicans* with 106 isolates (26%), while *C. dubliniensis* was the least isolated, with 3 isolates (0.4%). These findings align with the study by Umamaheshwari S et al. in South India, where *C. tropicalis* was also predominantly isolated, followed by *C. albicans*, and *C. dubliniensis* and *C. rugosa* were the least isolated. Another study by Lata R Patel et al. in North India also reported *C. tropicalis* as the most frequently isolated species, with *C. glabrata* being the least isolated (Jeddy et al., 2011; Shaik et al., 2016). The variation in *Candida* species distribution underscores the influence of geographical and demographic factors on the prevalence of different *Candida* species. This geographical variability is further highlighted by studies conducted in other regions. In a study conducted by Elias Seyoum et al. in Ethiopia, *C. albicans* was predominantly isolated, followed by *C. krusei.* Similarly, a study by Ga-Yeon Kim et al. in South Korea predominantly isolated *C. albicans*, while *C. parapsilosis* was the least isolated species (Jeddy et al., 2011; Kim et al., 2016).

The prevalence of fluconazole resistance among *Candida* species was 7.07% (29/410) in the current study. *C. tropicalis* was the most predominant isolate showing resistance to fluconazole, accounting for 51.72% (15/29) of the resistant cases. These findings align with the study conducted by Umamaheshwari S et al. in South India, where the fluconazole resistance rate was reported at 9.1%. Furthermore, a study conducted by Nadeen Jeddy et al. in Chennai, South India, reported a slightly higher fluconazole resistance rate of 10.5%, which might be attributed to local healthcare practices. Another study by Yonghao Xu et al. in China reported a fluconazole resistance percentage of 8.5%, indicating a similar trend. In their study, *C. albicans* contributed majorly to fluconazole resistance, possibly due to geographical variations in *Candida* species distribution and antifungal susceptibility patterns​ (Xu et al., 2008; Chongtham et al., 2022). On comparing this particular finding to Western literature, we found that the fluconazole resistance rate in *C. tropicalis* is lower. This might be due to the lower rate of *C. tropicalis* isolation in European countries, where *C. albicans* infections are predominant (Castanheira et al., 2020).

According to our study, 6 out of 15 fluconazole-resistant *C. tropicalis* isolates exhibited ERG11 mutation, accounting for a rate of 40%. This mutation is one of the key mechanisms leading to fluconazole resistance, as it involves alterations in the target enzyme lanosterol 14α-demethylase, reducing the drug’s binding efficacy​. Similar findings were reported in North India by Saikat Paul et al., where their study reported an ERG11 mutation rate of 37.5%. Another study conducted in South India by Kavitha M. K. et al. found a slightly higher mutation rate of 48.6%, suggesting regional variations in the prevalence of this resistance mechanism​. In contrast to our findings, a study conducted by Dan Wang et al. in China reported an ERG11 mutation rate of only 6.27%, indicating significant geographical differences in the prevalence of this genetic mutation​ (Wang et al., 2021; Paul et al., 2022; Kavitha and Arun, 2023). The A395T and C461T mutations in the ERG11 gene are associated with a high-level minimum inhibitory concentration (MIC) of azoles. These mutations are always found together, and no studies have reported the presence of either A395T or C461T independently. Additionally, ERG11 mutations are most commonly observed in *Candida albicans* and *Candida tropicalis* isolates.

In the present study, a discrepancy was observed between phenotypic fluconazole resistance detection and molecular characterization of ERG11 mutations among *C. tropicalis* isolates. While Vitek-2 and disk diffusion methods identified 15 and 13 fluconazole-resistant isolates, respectively, only 6 isolates were positive for the targeted ERG11 mutations (A395T and C461T) by PCR-HRM analysis (Table IV). This difference may be attributable to the presence of alternative resistance mechanisms that were not assessed in this study. Fluconazole resistance in Candida species can involve multiple molecular pathways beyond ERG11 gene mutations, including overexpression of efflux pumps (e.g., CDR1, MDR1), alterations in ergosterol biosynthesis genes other than ERG11, and biofilm formation, all of which can contribute to azole resistance phenotypes (Wang et al., 2021; Paul et al., 2022; Kavitha and Arun, 2023).

In addition, the PCR assay targeted only two specific mutations within the ERG11 gene; thus, isolates with other mutations or mechanisms conferring resistance may have been missed. This targeted approach, while useful for detecting known mutations, cannot capture the full molecular diversity of resistance. Moreover, while every effort was made to design specific and optimized primers, the possibility of suboptimal assay sensitivity or specificity cannot be completely excluded, especially without sequencing confirmation of PCR products. This limitation underscores the need for further molecular investigations using broader genomic approaches or sequencing for comprehensive resistance profiling. Therefore, the observed discrepancies likely reflect both the complex and multifactorial nature of fluconazole resistance in *Candida* and inherent limitations of the focused molecular assay employed in this study. Future studies incorporating whole-genome sequencing or additional molecular targets are warranted to better understand and correlate phenotypic resistance with underlying genetic mechanisms.

The study’s findings highlight the complexities of *Candida* infection patterns and resistance mechanisms. The significant prevalence of ERG11 mutations among fluconazole-resistant *C. tropicalis* isolates underscores the critical role of genetic mutations in antifungal resistance. This mutation, which affects the enzyme responsible for drug binding, reveals a key pathway through which *Candida* species develop resistance to azoles. The observed regional variations in mutation rates and resistance patterns emphasize the need for localized surveillance to adapt treatment strategies effectively. Continued research into these genetic mechanisms will be essential for advancing our understanding and management of *Candida* infections.

## Conclusion

The study highlights the efficacy and practicality of the Vitek2 system for both identifying *Candida* species and performing antifungal susceptibility testing, owing to its ease of use and cost-effectiveness. The disc diffusion method remains a valuable tool, especially in resource-limited settings, for guiding treatment decisions. Meanwhile, PCR and other molecular techniques offer significant research potential, providing deeper insights into the genetic mechanisms and mutations associated with fluconazole resistance. As fluconazole continues to be a critical antifungal treatment, understanding these resistance mechanisms is essential for improving patient outcomes and tailoring effective treatment strategies. Future research should focus on expanding the range of identified mutations and resistance mechanisms to better inform diagnostic and therapeutic approaches, ensuring robust management of *Candida* infections.

## Data Availability

The original contributions presented in the study are included in the article/supplementary material. Further inquiries can be directed to the corresponding author.

## References

[B1] Bart-Delabesse E. Boiron P. Carlotti A. Dupont B. (1993). Candida albicans genotyping in studies with patients with AIDS developing resistance to fluconazole. J. Clin. Microbiol. 31, 2933–2937. doi: 10.1128/jcm.31.11.2933-2937.1993, PMID: 7903316 PMC266158

[B2] Castanheira M. Deshpande L. M. Messer S. A. Rhomberg P. R. Pfaller M. A. (2020). Analysis of global antifungal surveillance results reveals predominance of Erg11 Y132F alteration among azole-resistant Candida parapsilosis and Candida tropicalis and country-specific isolate dissemination. Int. J. antimicrobial agents. 55, 105799. doi: 10.1016/j.ijantimicag.2019.09.003, PMID: 31520783

[B3] Chongtham U. R. Athokpam D. C. Singh R. A. (2022). Isolation, identification and antifungal susceptibility testing of Candida species: A cross-sectional study from Manipur, India. J. Clin. Diagn. Res. 16, 9–14. doi: 10.7860/JCDR/2022/55695.16248

[B4] Cilo B. D. Ener B. (2021). Comparison of clinical laboratory standards institute (CLSI) microdilution method and VITEK 2 automated antifungal susceptibility system for the determination of antifungal susceptibility of Candida species. Cureus 13, 1–8. doi: 10.7759/cureus.20220, PMID: 35004039 PMC8733416

[B5] Fadda M. E. Podda G. S. Pisano M. B. Deplano M. Viale S. Corda A. . (2010). Caratterizzazione fenotipica e molecolare di Candida isolate in un reparto di Terapia Intensiva [Phenotypic and molecular characterization of Candida species in ICU]. Ann Ig 22 (1), 9–17. Italian. Available online at: https://pubmed.ncbi.nlm.nih.gov/20476659/ (Accessed December 24, 2025). 20476659

[B6] Jeddy N. Ranganathan K. Uma Devi E. J. (2011). A study of antifungal drug sensitivity of Candida isolated from human immunodeficiency virus infected patients in Chennai, South India. J. Oral. Maxillofac. pathology: JOMFP. 15, 182. doi: 10.4103/0973-029X.84490, PMID: 22529577 PMC3329697

[B7] Kavitha M. K. Arun B. (2023). Molecular determination of ERG11 gene in fluconazole resistant strains of Candida tropicalis. World J Pharm Res. 12 (4), 119–125.

[B8] Kim G. Y. Jeon J. S. Kim J. K. (2016). Isolation frequency characteristics of Candida species from clinical specimens. Mycobiology. 44, 99–104. doi: 10.5941/MYCO.2016.44.2.99, PMID: 27433120 PMC4945544

[B9] Marak M. B. Dhanashree B. (2018). Antifungal susceptibility and biofilm production of Candida spp. isolated from clinical samples. Int. J. Microbiol. 2018, 7495218. doi: 10.1155/2018/7495218, PMID: 30405717 PMC6199855

[B10] Nnadi N. E. Ayanbimpe G. M. Scordino F. Okolo M. O. Enweani I. B. Criseo G. . (2012). Isolation and molecular characterization of Candida africana from Jos, Nigeria. Med. mycology. 50, 765–767. doi: 10.3109/13693786.2012.662598, PMID: 22380533

[B11] Patel L. R. Pethani J. D. Bhatia P. Rathod S. D. Shah P. D. (2012). Prevalence of Candida infection and its antifungal susceptibility pattern in tertiary care hospital, Ahmedabad. Natl. J. Med. Res. 2, 439–441. Available online at: https://njmr.in/index.php/file/article/view/835/745 (Accessed December 24, 2025).

[B12] Paul S. Dadwal R. Singh S. Shaw D. Chakrabarti A. Rudramurthy S. M. . (2021). Rapid detection of ERG11 polymorphism associated with azole resistance in Candida tropicalis. PloS One 16, e0245160. doi: 10.1371/journal.pone.0245160, PMID: 33439909 PMC7806177

[B13] Paul S. Shaw D. Joshi H. Singh S. Chakrabarti A. Rudramurthy S. M. . (2022). Mechanisms of azole antifungal resistance in clinical isolates of Candida tropicalis. PloS One 17, e0269721. doi: 10.1371/journal.pone.0269721, PMID: 35819969 PMC9275685

[B14] S U. Sumana M. N. (2023). Retrospective analysis on distribution and antifungal susceptibility profile of Candida in clinical samples: a study from Southern India. Front. Public Health 11. doi: 10.3389/fpubh.2023.1160841, PMID: 37261242 PMC10228385

[B15] Sailaja B. Prasad P. (2019). A study on isolation of Candida species in various clinical samples in a tertiary health care unit. Indian J. Microbiol. Res. 6, 258–260. doi: 10.18231/j.ijmr.2019.056

[B16] Sandven P. (1999). Detection of fluconazole-resistant Candida strains by a disc diffusion screening test. J. Clin. Microbiol. 37, 3856–3859. doi: 10.1128/JCM.37.12.3856-3859.1999, PMID: 10565896 PMC85829

[B17] Sardari A. Zarrinfar H. Mohammadi R. (2019). Detection of ERG11 point mutations in Iranian fluconazole-resistant Candida albicans isolates. Curr. Med. Mycology. 5, 7. doi: 10.18502/cmm.5.1.531, PMID: 31049452 PMC6488286

[B18] Sem J. N. Morubagal R. R. Shivappa S. G. Gowda R. S. Mahale R. P. Urs T. A. . (2024). Recent trends in the Susceptibility pattern of Candida to Fluconazole and Amphotericin B at a tertiary care centre in South India. IP Int. J. Med. Microbiol. Trop. Dis. 10, 62–66. doi: 10.18231/j.ijmmtd.2024.011

[B19] Seyoum E. Bitew A. Mihret A. (2020). Distribution of Candida albicans and non-albicans Candida species isolated in different clinical samples and their *in vitro* antifungal susceptibility profile in Ethiopia. BMC Infect. Diseases. 20, 1–9. doi: 10.1186/s12879-020-4883-5, PMID: 32188422 PMC7081544

[B20] Shaik N. Penmetcha U. Myneni R. B. Yarlagadda P. Singamsetty S. (2016). A study of identification and antifungal susceptibility pattern of Candida species isolated from various clinical specimens in a tertiary care teaching hospital, Chinakakani, Guntur, Andhra Pradesh, South India. Int. J. Curr. Microbiol. App Sci. 5, 71–91. doi: 10.20546/ijcmas.2016.507.006

[B21] Tan J. Zhang J. Chen W. Sun Y. Wan Z. Li R. . (2015). The A395T mutation in ERG11 gene confers fluconazole resistance in Candida tropicalis causing candidemia. Mycopathologia. 179, 213–218. doi: 10.1007/s11046-014-9831-8, PMID: 25398256

[B22] Vandeputte P. Larcher G. Berges T. Renier G. Chabasse D. Bouchara J. P. (2005). Mechanisms of azole resistance in a clinical isolate of Candida tropicalis. Antimicrobial Agents Chemotherapy. 49, 4608–4615. doi: 10.1128/AAC.49.11.4608-4615.2005, PMID: 16251302 PMC1280149

[B23] Vignesh Kanna B. Amar Kumar G. Swapna M. Joshy M. E. (2017). Isolation and identification of candida species from various clinical samples in a tertiary care hospital. Int. J. Res. Med. Sci. 5, 3520–3522. doi: 10.18203/2320-6012.ijrms20173554

[B24] Wang D. An N. Yang Y. Yang X. Fan Y. Feng J. (2021). Candida tropicalis distribution and drug resistance are correlated with ERG11 and UPC2 expression. Antimicrobial Resistance Infection Control. 10, 1–9. doi: 10.1186/s13756-021-00890-2, PMID: 33722286 PMC7958445

[B25] Wang M. Cao Y. Xia M. Al-Hatmi A. M. Ou W. Wang Y. . (2019). Virulence and antifungal susceptibility of microsatellite genotypes of Candida albicans from superficial and deep locations. Yeast. 36, 363–373. doi: 10.1002/yea.3397, PMID: 31037772 PMC6618086

[B26] Xu Y. Chen L. Li C. (2008). Susceptibility of clinical isolates of Candida species to fluconazole and detection of Candida albicans ERG11 mutations. J. Antimicrobial Chemotherapy. 61, 798–804. doi: 10.1093/jac/dkn015, PMID: 18218640

[B27] Ying C. Zhang H. Tang Z. Chen H. Gao J. Yue C. (2015). Antifungal susceptibility and molecular typing of 115 Candida albicans isolates obtained from vulvovaginal candidiasis patients in 3 Shanghai maternity hospitals. Sabouraudia. 54, 394–399. doi: 10.1093/mmy/myv082, PMID: 26468549

[B28] Ying Y. Zhao Y. Hu X. Cai Z. Liu X. Jin G. . (2013). *In vitro* fluconazole susceptibility of 1,903 clinical isolates of Candida albicans and the identification of ERG11 mutations. Microbial Drug Resistance. 19, 266–273. doi: 10.1089/mdr.2012.0204, PMID: 23484590

